# The Role of Rice HEI10 in the Formation of Meiotic Crossovers

**DOI:** 10.1371/journal.pgen.1002809

**Published:** 2012-07-05

**Authors:** Kejian Wang, Mo Wang, Ding Tang, Yi Shen, Chunbo Miao, Qing Hu, Tiegang Lu, Zhukuan Cheng

**Affiliations:** 1 State Key Laboratory of Plant Genomics and Center for Plant Gene Research, Institute of Genetics and Developmental Biology, Chinese Academy of Sciences, Beijing, China; 2 Biotechnology Research Institute/National Key Facility for Gene Resources and Gene Improvement, Chinese Academy of Agricultural Sciences, Beijing, China; University of Birmingham, United Kingdom

## Abstract

HEI10 was first described in human as a RING domain-containing protein that regulates cell cycle and cell invasion. Mice *HEI10^mei4^* mutant displays no obvious defect other than meiotic failure from an absence of chiasmata. In this study, we characterize rice *HEI10* by map-based cloning and explore its function during meiotic recombination. In the rice *hei10* mutant, chiasma frequency is markedly reduced, and those remaining chiasmata exhibit a random distribution among cells, suggesting possible involvement of HEI10 in the formation of interference-sensitive crossovers (COs). However, mutation of HEI10 does not affect early recombination events and synaptonemal complex (SC) formation. HEI10 protein displays a highly dynamic localization on the meiotic chromosomes. It initially appears as distinct foci and co-localizes with MER3. Thereafter, HEI10 signals elongate along the chromosomes and finally restrict to prominent foci that specially localize to chiasma sites. The linear HEI10 signals always localize on ZEP1 signals, indicating that HEI10 extends along the chromosome in the wake of synapsis. Together our results suggest that HEI10 is the homolog of budding yeast Zip3 and *Caenorhabditis elegans* ZHP-3, and may specifically promote class I CO formation through modification of various meiotic components.

## Author Summary

Meiosis is a specialized cell division that is essential for sexual reproduction. Errors in meiosis are the leading cause of both infertility and birth defects. Most meiotic genes and their functions seem to be highly conserved among different species. HEI10 was first identified to regulate cell cycle and cell invasion in human somatic cells. Studies in mice revealed that the mutation of *HEI10* led to the absence of chiasmata and a final sterile phenotype. However, the exact function of HEI10 in meiotic recombination remains unresolved. In this study, we characterize the rice HEI10 homolog and demonstrate that HEI10 is required for normal crossover formation, but not for synapsis. HEI10 protein initially appears as distinct foci. Thereafter, the signals elongate along the SC and finally restrict to prominent foci that correspond to future chiasma sites. In addition, we also show a close relationship of HEI10 with two other characterized ZMM proteins, ZEP1 and MER3. We propose that the HEI10 exerts its effects in the recombination process through modification of diverse meiotic components.

## Introduction

Reduction division is unique to eukaryotic cells undergoing meiosis. It requires specific mechanisms to pair the homologous chromosomes in their proper orientation and for correct separation [1]. In most organisms, a stable connection is established by crossovers (COs). COs not only ensure the proper homologous segregation, but create new combinations of alleles leading to increased variation [2]. The number and distribution of COs are tightly controlled. If multiple COs occur along a homologous pair, they are more evenly spaced than would be expected by chance. This phenomenon is known as interference. Interference-sensitive COs belong to the class I recombination pathway and account for most COs. In addition, some COs are not subject to interference, and these are referred to as class II interference-insensitive COs [3]–[5].

In budding yeast, the formation of class I COs is catalyzed by the recruitment of a set of meiosis-specific proteins collectively referred to as ZMM (for Zip1, Zip2, Zip3, Zip4, Msh4, Msh5 and Mer3). Co-localization of ZMM proteins and similarities among *zmm* mutant phenotypes suggest functional collaboration among these proteins [5]–[7]. Zip1 is the transverse filament component of the synaptonemal complex (SC) [8]. MER3 is a DNA helicase that unwinds various duplex DNA in an ATP-dependent manner [9]. MSH4 and MSH5 appear to function as a heterodimer to stabilize strand invasion [10]–[11]. Zip2 is related to WD40-like repeat protein [12] while Zip4 is a tetra-tricopeptide repeat (TPR) protein [13]. Zip3 is a RING domain-containing protein that may have ubiquitin or small ubiquitin-like modifier (SUMO) ligase activity [14]–[15]. Zip2, Zip3, and Zip4 proteins are implicated in ubiquitinylation and/or SUMOylation and likely work together to modify protein interactions [13], [16]–[17]. Zip3 was initially implicated in promoting synapsis [15]. However, a recent study also revealed that Zip3 inhibits SC assembly specifically at centromeres, where it is considered to be dispensable for promoting synapsis in budding yeast [18]–[19].

Several presumed ZMM homologs have been identified in plants, suggesting the conservation of ZMM group polypeptides among different species. MER3, MSH4 and MSH5 are all highly conserved proteins and their orthologs have been identified and functionally investigated in plants [20]–[24]. Despite of poor sequence conservation, Zip1 homologs, ZYP1 and ZEP1, have been isolated in *Arabidopsis* and rice, respectively. However, mutations of either ZYP1 or ZEP1 did not lead to obvious reduction in COs, indicating different roles of them during CO formation [25]–[26]. Presumed Zip4 orthologs have been identified in *Arabidopsis* and rice [27]–[28], and a recent study revealed that *Arabidopsis* SHOC1 is orthologous to Zip2 [29]–[30]. In addition, a novel gene, *PARTING DANCERS* (*PTD*), was found to be epistatic to ZMMs in *Arabidopsis* [31]. However, no Zip3 orthologs have been detected in plants. Until now, *C. elegans* ZHP-3 is the only identified ortholog of budding yeast Zip3. In homozygous *zhp-3* knockout worms, homolog pairing and SC formation occur normally, but chiasmata were completely absent, indicating an important role of ZHP-3 during reciprocal recombination [32]. Intriguingly, in contrast to budding yeast where Zip3 consistently localizes to distinct foci [15], *C. elegans* ZHP-3 showed various localization patterns at different stages [32], [33], suggesting probable divergent roles of ZHP-3 and Zip3 during meiosis. Different from most organisms, *C. elegans* is a multicelluar organism in which SC formation is independent of double-strand breaks (DSBs), and only class I COs seem to occur. Whether the functional conservation of Zip3 and ZHP-3 is universal in other higher organisms remains as yet unresolved and needs to be explored.

Human Enhancer of Invasion 10 (HEI10; also known as CCNB1IP1) was first identified in a functional genomic screen for novel human genes that influenced cell cycle progression and/or polarization [34]. HEI10 functions as an E3 Ubiquitin ligase to regulate cell migration and invasion [34], [35]. Studies in mice revealed that mutation of HEI10 led to dramatic meiosis defects, indicating an important role of HEI10 during meiosis. Furthermore, results obtained in experiments using a yeast two-hybrid system suggest a function for HEI10 as E3 SUMO ligase in addition to the ubiquitin ligase role reported in somatic cells [35]. It is further known that HEI10 is required for meiotic CO formation. Mechanistic linkage of HEI10 to recombination has yet to be established, partially because of the lack of localization experiments. Here, we investigated the role of HEI10 in rice and suggest that HEI10 might be the homolog of budding yeast Zip3 and *C. elegans* ZHP-3. However, those genes may play both conserved and divergent roles in homologous recombination in their respective species.

## Results

### Characterization of a sterile mutant in rice

We obtained a sterile mutant from the *japonica* rice variety *Wuxiangjing 9* which was induced by ^60^Co γ-ray irradiation. The mutant showed normal vegetative growth but exhibited complete sterility (Figure S1A and S1B). Cytological observation of anthers showed that almost all pollens were shrunken and inviable (Figure S1C and S1D). When pollinated with wild-type (WT) pollens, the mutant spikelets were unable to set any seeds, suggesting that the female gametes were also sterile. The progenies of the heterozygous plants segregated from normal to sterile phenotype in a 3∶1 ratio (fertile, 30; sterile, 10), indicating that a single recessive gene is responsible for the sterile phenotype.

### Molecular cloning of the *HEI10* gene

**Figure 1:**
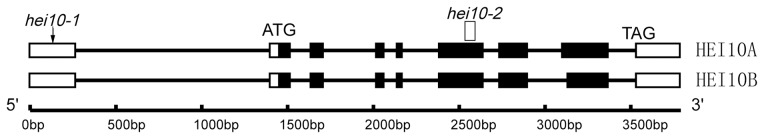
Organization of the *HEI10* gene and protein alignment. (A) Schematic representation of *HEI10* gene and the location of *hei10* mutation. Translated regions are represented by black boxes and untranslated regions are indicated by white boxes. HEI10A and HEI10B indicate long and short transcripts, respectively.

To isolate the mutated gene that controls the sterile phenotype, we began by isolating the gene through map-based cloning. A large F2 mapping population was generated by crossing the heterozygous plants with the *indica* rice variety 9311. Using sterile plants that segregated in F2 and F3 populations, we mapped the gene on the long arm of rice chromosome 2, which was further narrowed to a 100-kb region. All genes within this region were amplified and sequenced. A single nucleotide G to A substitution was found at position 140 of the first exon of the *Os02g0232100* gene (Figure 1). This substitution introduced a new translation initiation site (ATG) in the 5′-UTR, which would theoretically express a totally different peptide. We named the mutant *hei10-1* based on the homology of the protein sequence (see below).

We also obtained another mutant showing the same cytological defects. It was obtained from a *japonica* cultivar Nipponbare, induced by tissue culture. Map-based cloning showed that the mutated gene was located in the same region. Sequencing of the *Os02g0232100* gene of the mutant revealed a 59 bp deletion in the sixth exon, which resulted in a premature stop codon at amino acid residue 126. Thus, this mutant was designated *hei10-2* mutant.

By means of RT-PCR on young panicles, we isolated two *HEI10* cDNAs, which were caused by an alternative splicing in the eighth exon. The two cDNAs encoded a 304- and a 295-amino acid (aa) protein, respectively (Figure S2). The larger protein (HEI10A) had nine extra aa at position 214 compared to the short protein (HEI10B). Using the HEI10A protein sequence, we performed a BLAST search in public databases and revealed that rice HEI10A shares significant similarity with HEI10/CCNBP1IP1 protein of human (26% identity and 51% similarity over a 179-aa region) and mouse (25% identity and 50% similarity over a 179-aa region) origin. Reciprocal BLAST searches further confirmed that the isolated protein is the closest relative of mammalian HEI10 in rice, leading us to name the isolated gene *HEI10*. In addition, a search for a homolog in the *C. elegans* database revealed that ZHP-3 protein is the closest homolog of HEI10A in *C. elegans* (27% identity and 43% similarity over a 169-aa region). Reciprocal BLAST searches in the rice database revealed that HEI10 is the closest relative of ZHP-3. As ZHP-3 represents the homolog of budding yeast Zip3, we further performed a sequence alignment using HEI10A and Zip3 sequences and found some similarity between them (25% identity and 38% similarity over a 126-aa region). A search in SMART for conserved domains revealed two domains in the HEI10A protein, a RING domain in the N terminus region (aa residues 3–42) and a coiled coil region (aa residues 119–184). The two domains were conserved in all obtained HEI10 orthologues. Taken together, it seems that HEI10 is the most likely homologue of Zip3 and ZHP-3.

### Chromosome behavior in *hei10* mutants

To find out whether and, if so, how mutations in *HEI10* affect meiosis, the meiotic chromosomes of pollen mother cells (PMCs) at different stages of meiotic division from both WT rice and *hei10* mutants were investigated by staining meiotic chromosome spreads with 4′,6-diamidino-2-phenylindole (DAPI). In WT, the chromosomes started to condense and could be seen as thin threads at the leptotene stage. At zygotene, homologous chromosomes underwent pairing and synapsis initiation events. Synapsis was completed, and fully synapsed chromosomes were visible at pachytene (Figure S3A). After further condensation during the diplotene phase, 12 highly condensed bivalent chromosomes were clearly observed at diakinesis (Figure S3B). At metaphase I, all bivalents aligned in the center of the cell (Figure S3C). Afterwards, homologous chromosomes separated at anaphase I, generating dyads (Figure S3D and S3E). During meiosis II, the two dyads divided simultaneously and produced tetrads (Figure S3F).

**Figure 2:**
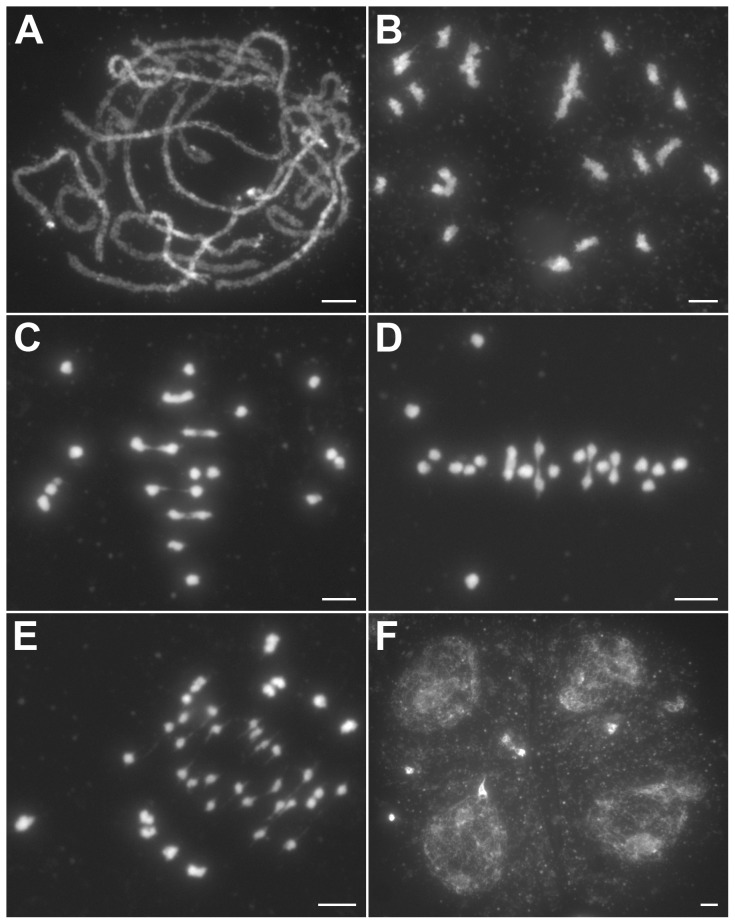
Meiosis in the *hei10-1* mutant. (A) Pachytene. (B) Diakinesis with both bivalents and univalents. (C) Metaphase I with five bivalents and fourteen univalents. (D) Metaphase I with four bivalents and sixteen univalents. Thirteen univalents and all bivalents align on the equatorial plate. (E) Anaphase I. Many univalents undergo a precocious segregation of sister chromatids. (F) Telophase II. Tetrads with several randomly distributed chromosomes. Chromosomes were stained with DAPI. Scale bars, 5 µm.

The *hei10* mutant chromosomes behaved normally during leptotene and zygotene. Fully aligned chromosomes were detected during pachytene (Figure 2A). However, during diakinesis, the mutant cells showed a mixture of both univalent and bivalent chromosomes (Figure 2B). At metaphase I, the bivalents aligned well on the equatorial plate while some of the univalents were scattered in the nucleus (Figure 2C). In anaphase I, the bivalents separated normally but the scattered univalents segregated randomly. Besides those randomly distributed univalents, many univalents also aligned on the equatorial plate in metaphase I (Figure 2D) and underwent precocious separation of sister chromatids in anaphase I (Figure 2E), indicating a bipolar orientation of sister kinetochores. In telophase I and prophase II, an uneven number of chromosomes was observed in the two related cells. After the second division, tetrads with aberrant numbers of chromosomes were formed. In addition, multiple micronuclei were frequently observed (Figure 2F).

**Figure 3:**
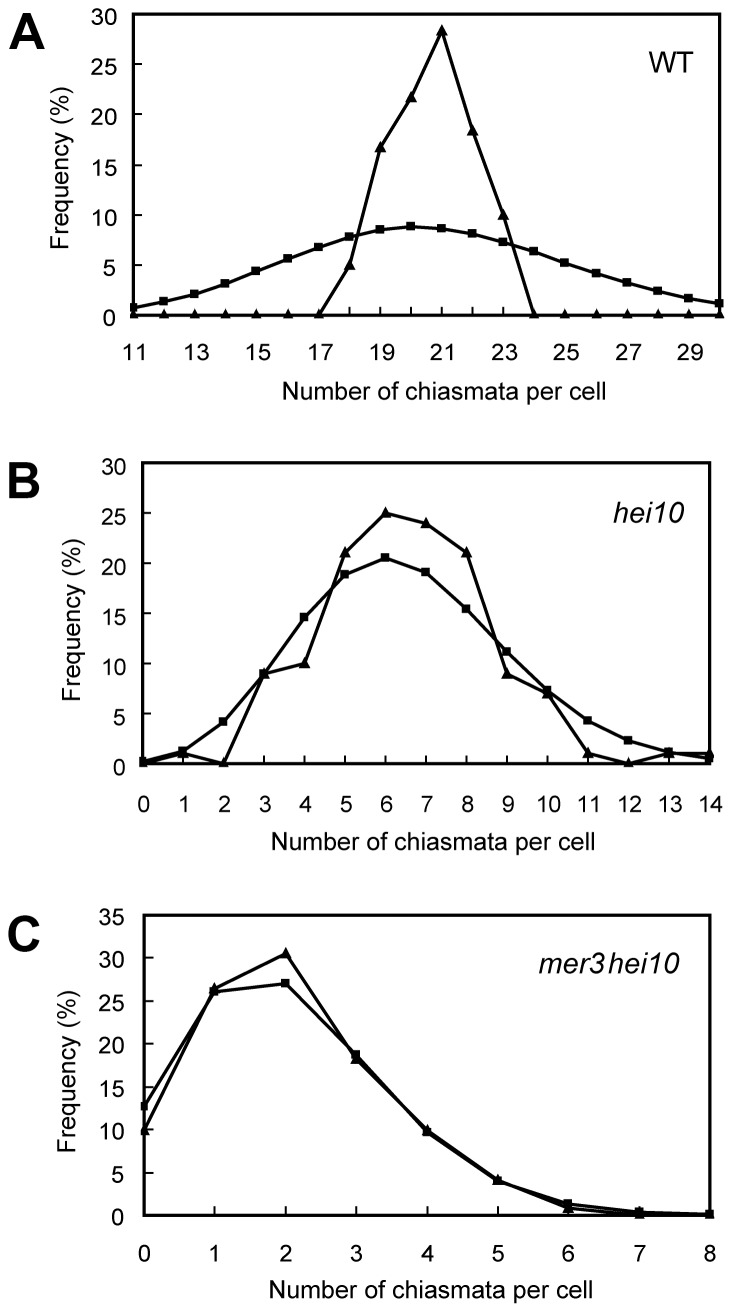
Chiasma distribution in WT, *hei10*, and *mer3hei10*. (A) Chiasma distribution in the WT. The observed distribution of chiasmata deviates from a Poisson distribution. (B) In *hei10*, the observed distribution of chiasmata is consistent with a Poisson distribution. (C) In *mer3hei10*, the observed distribution of chiasmata is consistent with a Poisson distribution. Triangles indicate observed distribution, whereas squares show predicted Poisson distribution.

The number of chiasmata in *hei10-1* and WT was quantified by studying the shape of metaphase I bivalents using criteria described previously [36]. The rod-shaped and ring-shaped bivalents were treated as having one and two chiasmata, respectively. For example, in Figure S3C, the cell contained two bivalents with only one chiasma and ten bivalents with two chiasmata. The chiasma frequency in WT was 20.7±1.4 per cell while in *hei10-1* mutant the chiasma frequency was 6.5±2.1 per cell, corresponding to 5.4 bivalents per PMC (n = 130). Thus, the mutation of *hei10* led to a significant reduction in chiasma frequency and number of bivalent chromosomes. In WT, the distribution of chiasmata deviated from a Poisson distribution among different PMCs (*χ*_[23]_^2^ = 65.7, *P*<0.01) (Figure 3A). In contrast, chiasma frequency per cell in *hei10* mutants was consistent with a Poisson distribution (*χ*_[14]_^2^ = 16.0, *P*>0.1) (Figure 3B) suggesting that the majority of residual chiasmata are randomly distributed among cells.

In order to further determine the role of HEI10 in CO formation, we generated the *mer3hei10* double mutant and compared the chiasma number to each single mutant. In the *mer3hei10* double mutant, the chiasma frequency was 2.1±1.3 per cell (n = 121). The mean number of chiasmata of *mer3hei10* double mutant was significantly reduced compared with either *hei10* mutant (*t*_[249]_ = 19.9, P<0.01) or *mer3* mutant (*t*_[202]_ = 15.4, P<0.01). In addition, the remaining chiasmata also followed a Poisson distribution (*χ*_[6]_^2^ = 1.3, *P*>0.1) (Figure 3C), indicating those residual chiasmata are also distributed randomly among cells.

### REC8, MER3, and ZEP1 localize normally in *hei10* mutants

**Figure 4:**
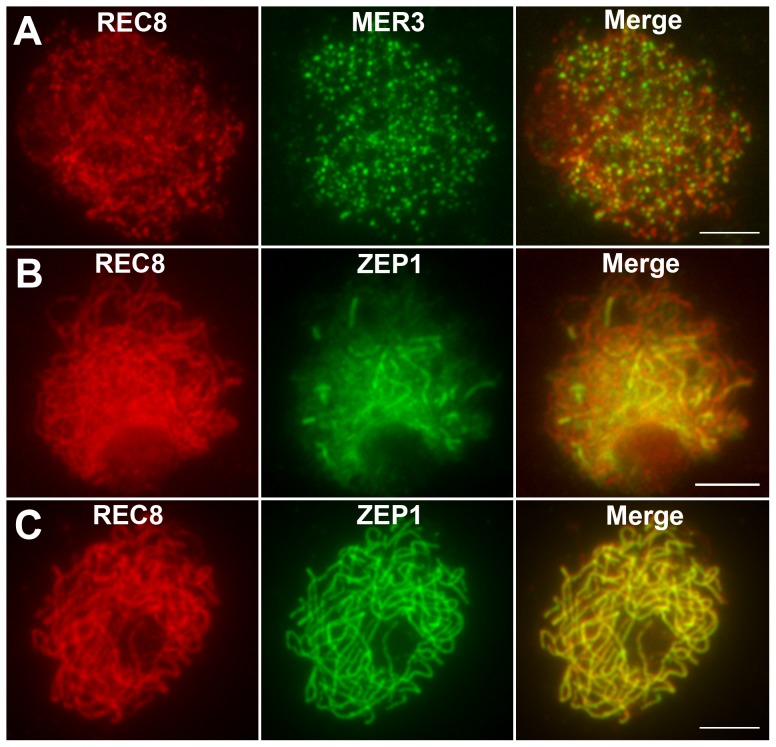
Immunolocalization of REC8, MER3, and ZEP1 in *hei10-1*. (A) The localization of REC8 and MER3 at late leptotene. (B) The localization of REC8 and ZEP1 at zygotene. (C) The localization of REC8 and ZEP1 at pachytene. Scale bars, 5 µm.

REC8 is a component of the cohesion complex and is required for sister chromatid cohesion, axial element formation, and homolog pairing. Its signals can be used to indicate assembly of cohesion complex in rice [37]. In WT, REC8 was first detected as punctuate foci at premeiotic interphase. At late leptotene, these foci assembled to form short linear signals. REC8 appeared as thin threads along the chromosomes in early zygotene and became thicker thereafter. From interphase to pachytene, the localization of REC8 in the *hei10* mutant was similar to the localization observed in WT (Figure 4A–4C). The rice MER3 protein is required for the formation of COs. Knowing the number and localization of MER3 foci may give an indication of a potential defect at early steps in the crossover pathway [20]. In both WT and *hei10-1*, MER3 appeared as foci on chromosomes in early leptotene (Figure 4A). In WT, the mean number of MER3 foci at late leptotene was 296.7 (range 243–331, n = 10) which reduced to 140.1 (range 111–164, n = 6) at early pachytene. Similarly, the mean number of MER3 foci in *hei10-1* was 307.8 (range 278–339, n = 10) at late leptotene and decreased to 138.2 (range 115–163, n = 8) at early pachytene. So, the normal turnover of MER3 in *hei10* suggested that the early processing of recombination intermediates is not disrupted. In addition, as the reduced number of COs is not compatible with the appearance of broken chromosomes, it seems that most of those recombination intermediates in *hei10* are eventually repaired as NCOs or resolved toward the sister chromatid.

The DAPI-stained chromosomes indicated that the homologs paired well in *hei10-1* mutants. To elucidate the effect of the *hei10-1* mutation on synapsis, dual immunolocalization was performed using antibodies against REC8 and ZEP1. ZEP1 is a component of the SC central element and its distribution indicates the extent of synapsis in rice [25]. In *hei10* mutants, ZEP1 appeared as linear signals at zygotene (Figure 4B), indicating that SC polymerization is not obviously affected. At pachytene, ZEP1 formed long linear signals along the whole length of the chromosomes (Figure 4C). Overall, we did not detect obvious ZEP1 localization abnormalities in *hei10* mutant. Therefore, the indistinguishable localization pattern of ZEP1 in *hei10* and WT suggests that HEI10 may not be required for synapsis to proceed.

### HEI10 shows a dynamic localization pattern

To accurately define the spatial and temporal distribution of HEI10 during meiosis in rice, dual immunolocalization experiments were performed using polyclonal antibodies against REC8 and HEI10 protein, raised in rabbit and mouse, respectively.

**Figure 5:**
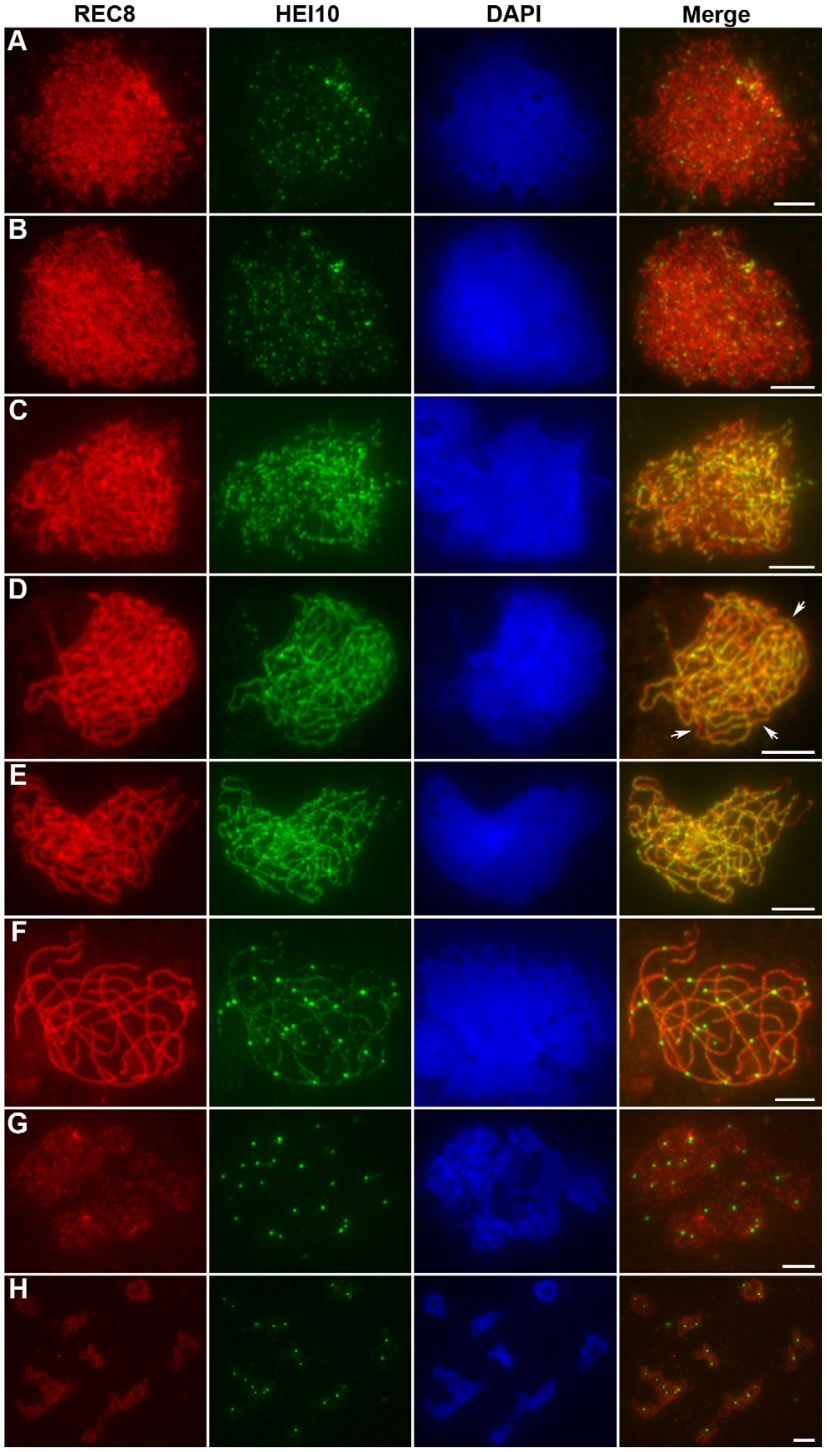
Immunolocalization of REC8 and HEI10 in WT rice. (A) Early leptotene, HEI10 appears as distinct foci. (B) Late leptotene. (C) Zygotene. (D) Late zygotene/Early pachytene, arrows indicate the gaps of HEI10 linear signals. (E) Middle pachytene. (F) Late pachytene, prominent foci localize on the chromosomes. (G) Diplotene. (H) Diakinesis, HEI10 foci locate at the chiasmata position. Scale bars, 5 µm.

**Figure 6:**
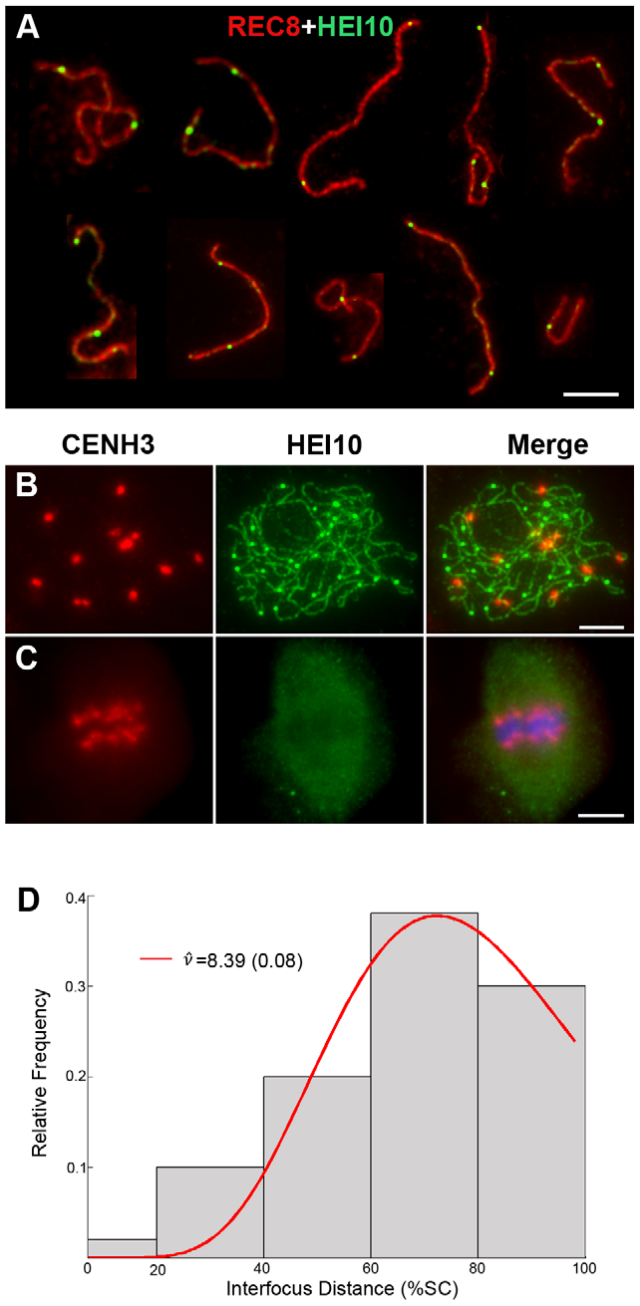
Analysis of the distribution of HEI10 bright foci in WT meiocytes. (A) Dual immunolocalization of WT scattered chromosomes with REC8 (red) and HEI10 (green) antibodies. Scale bar: 5 µm. (B) Dual immunolocalization of CENH3 and HEI10 at late pachytene. (C) No HEI10 signals were detected at metaphase I, chromosomes are stained with DAPI (blue). Scale bars: 5 µm. (D) Histogram of the observed distances between two adjacent HEI10 bright foci on the shortest chromosomes (percentages of the length of the synaptonemal complex). The gray bars show the observed relative frequencies of inter-HEI10 foci distances. The red curve presents the best fit of the distance to the gamma distribution. *ν* is the interference parameter in the gamma model. 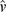 is the *ν* value (estimated SE) for which the best fit of the observed distances to the gamma model was got. Here, the estimated 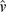 is 8.39, which indicates a strong interference among HEI10 bright foci on the shortest chromosome in rice (if there is no interference, *ν* is 1).

HEI10 was first visible as punctuated foci at early leptotene and almost all of them localized on REC8 signals (Figure 5A). The number of foci increased rapidly at leptotene. The mean number of HEI10 foci at late leptotene per nucleus was 297.1 (n = 15, range 223–340) (Figure 5B). Apart from distinct foci, during zygotene linear arrays of dots or short lines were also found (Figure 5C). At late zygotene/early pachytene, the linear signals extended along entire chromosomes. However, discontinuities or gaps were obvious in HEI10 linear signals (Figure 5D). In mid-pachytene, the discontinuities/gaps were even more apparent and most linear signals were split into linear arrays of dots (Figure 5E). At late pachytene, some bright foci appeared on each chromosome, but faint linear signals and foci were still observed along the chromosomes in most meiocytes (Figure 5F). The average number of HEI10 faint foci and bright foci per cell was 284.9 (n = 21, range 204–352) and 24.5 (n = 21, range 21–29) respectively. At diplotene, all other faint signals disappeared and only those bright foci remained (average 24.7, n = 32, range 20–28; Figure 5G). At diakinesis, a mean of 24.3 (n = 22, range 21–30) foci was detected. Furthermore, we found that those bright HEI10 foci located at the chiasma position (Figure 5H). By metaphase I, no HEI10 signals were detected (Figure 6C).

To obtain the precise localization of HEI10 bright foci on the chromosomes, we focused on the foci positions at late pachytene. Observation of scattered chromosomes revealed that one, two or three, frequently two, prominent foci localized on each pair of homologs (Figure 6A). Additionally, when two or three foci occurred on the same chromosome, they tended to be spaced far apart. Immunostaining using antibodies against HEI10 and CENH3 also revealed that about 95.6% HEI10 foci located outside CENH3 position at late pachytene (Figure 6B). To further explore whether those HEI10 bright foci were randomly distributed along bivalents, we measured the interfocus distance among bright HEI10 focus on the shortest chromosome of the cell and estimated the existence of interference using the interference parameter *ν* of the gamma model [38], [39] (Figure 6D). The result showed that HEI10 bright foci on a single chromosome displayed strong interference.

### Co-localization between MER3 and HEI10

**Figure 7:**
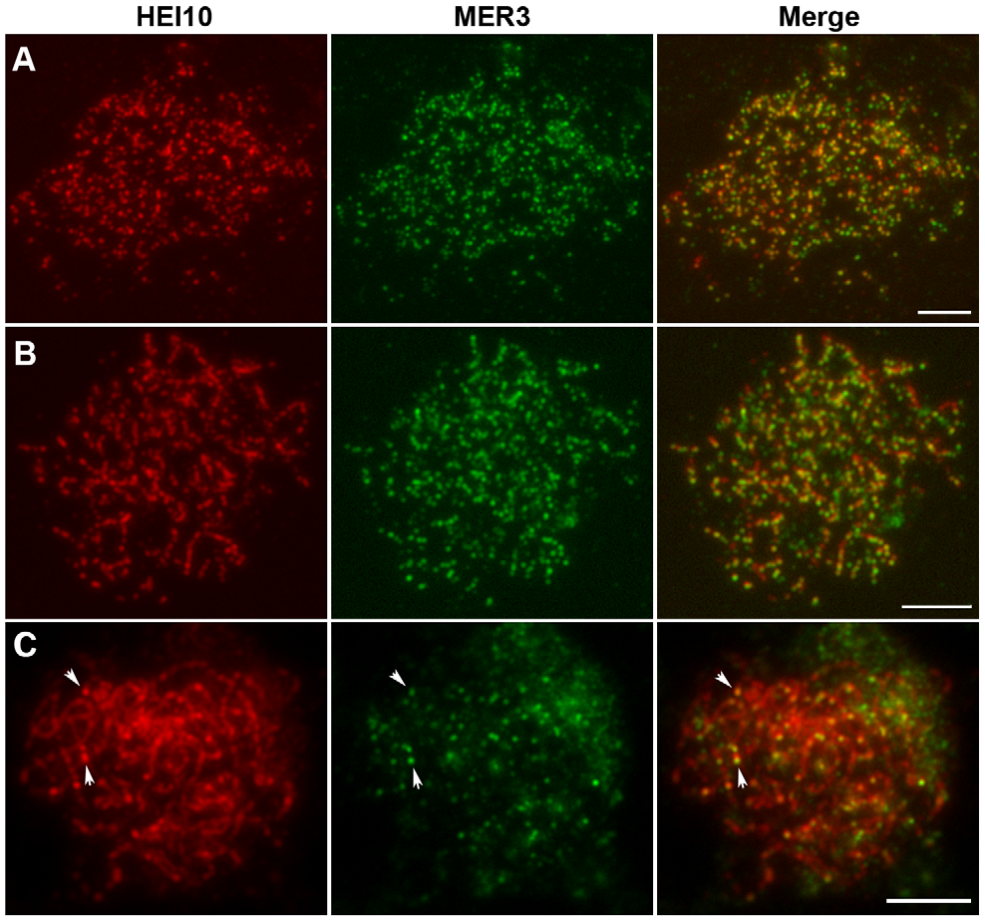
Dual immunolocalization of HEI10 and MER3 in WT meiocytes. (A) Leptotene, HEI10 foci and MER3 foci show a high co-localization. (B) Zygotene, MER3 foci localize on HEI10 foci or short stretches. (C) Pachytene. Arrows indicate the co-localization of HEI10 foci and remaining MER3 foci. Scale bars, 5 µm.

Previous studies noted punctuate foci of MER3 protein in early prophase I. To establish the relationship between HEI10 and MER3, we performed dual immunostaining experiments using antibodies against HEI10 and MER3, raised in rabbits and mice, respectively. As shown in Figure 7A, discrete foci of HEI10 almost completely co-localized with foci of MER3 in the leptotene stage (93.1% of MER3 foci contain HEI10, 97.5% of HEI10 foci contain MER3). At zygotene, HEI10 began to elongate along the chromosomes and still showed high co-localization with MER3 foci. Most MER3 foci localized on the linear HEI10 signals and almost all HEI10 short stretches had at least one MER3 foci (Figure 7B). During pachytene, MER3 foci decreased rapidly but all those remaining foci still localized on HEI10 linear signals or emerged bright foci (Figure 7C). At late pachytene, prominent HEI10 foci appeared and persisted to later stages, whereas MER3 foci were not visible any more.

### Co-localization between HEI10 and ZEP1

**Figure 8:**
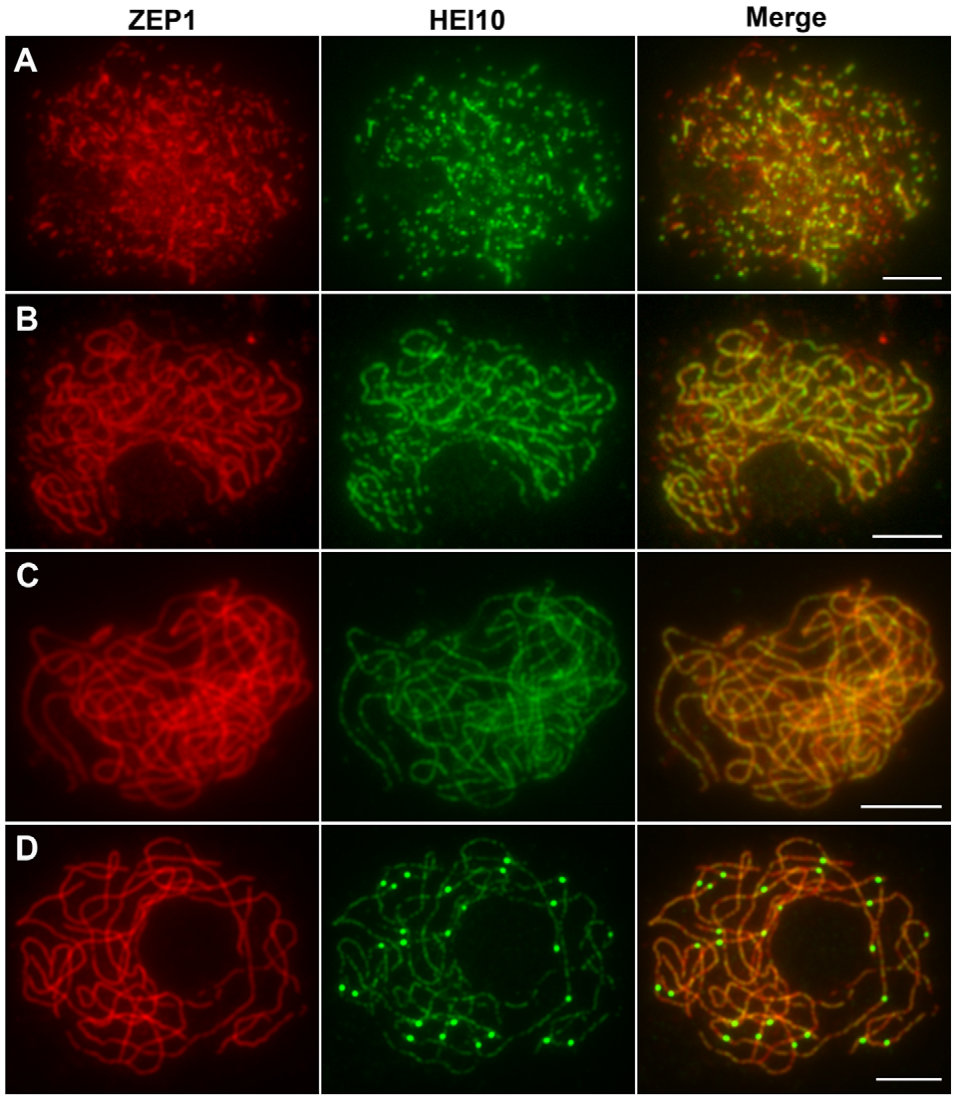
Dual immunolocalization of HEI10 and ZEP1 in WT meiocytes. (A) Early zygotene. (B) Late zygotene, HEI10 signals localize on ZEP1 linear signals. (C) Early pachytene, HEI10 signals overlap well with ZEP1 signals. (D) Late pachytene, prominent HEI10 foci localize on ZEP1 linear signals. Scale bars, 5 µm.

The localization of HEI10 proteins during zygotene and early pachytene appeared similar to that of ZEP1 proteins, thus, we further investigated the co-localization between these proteins using their respective antibodies. At early zygotene, when both HEI10 and ZEP1 formed linear signals, there was extensive co-localization between them (Figure 8A). At late zygotene, the co-localization was even more apparent (Figure 8B). While HEI10 was always present on chromosomal segments that stained with anti-ZEP1 antibodies, HEI10 signals did not always co-localize with ZEP1. Overall, we rarely observed HEI10 linear signals outside ZEP1-positive signals. These results suggest that localization of ZEP1 to chromosomes normally precedes HEI10 extension. During early pachytene, HEI10 signals, except for some discontinuities, overlapped well with ZEP1 signals (Figure 8C). At late pachytene, some prominent bright HEI10 foci appeared on chromosomes, and these signals localized on ZEP1 linear signals (Figure 8D).

### HEI10 localization in *pair3*, *zep1*, and *mer3* mutants

**Figure 9:**
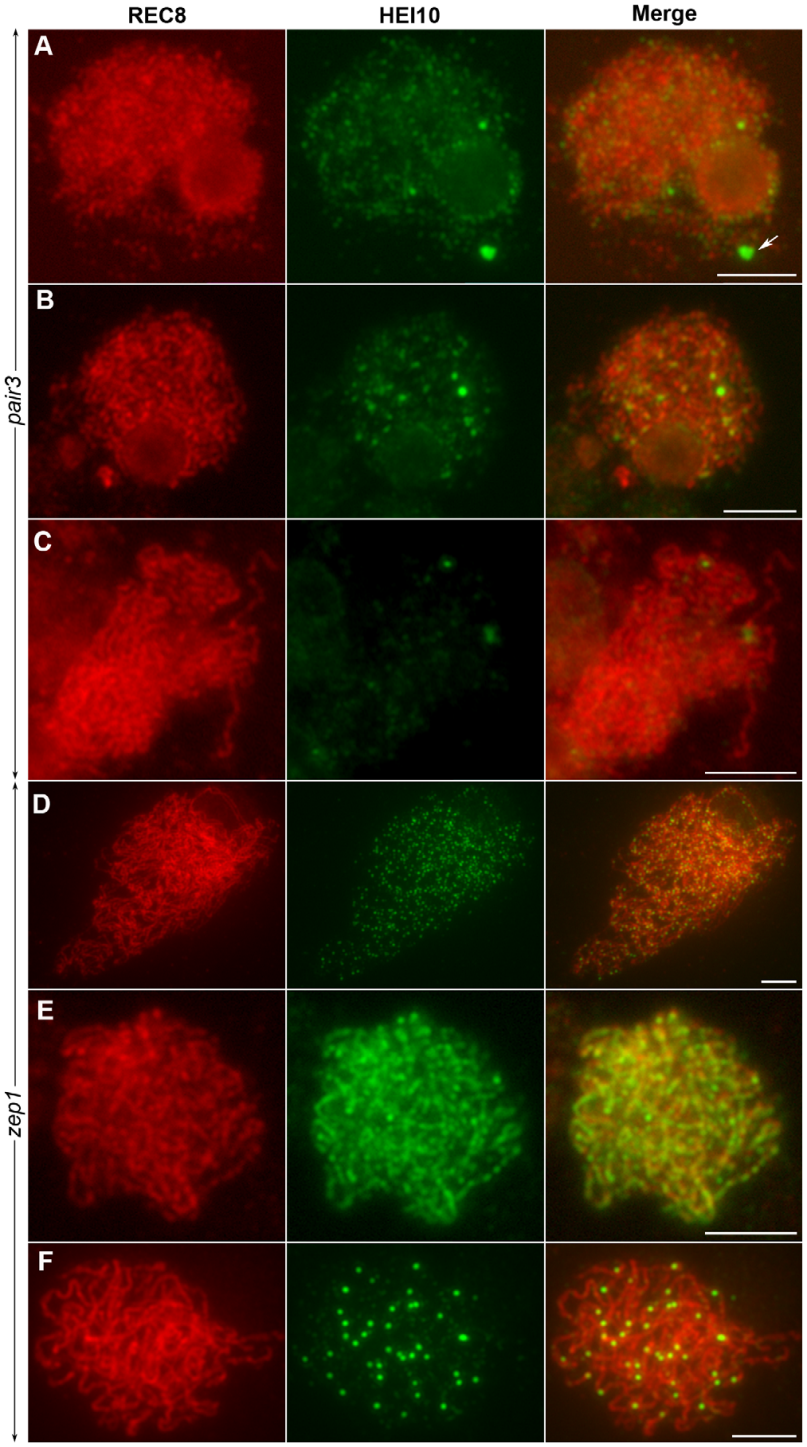
The localization of HEI10 in *pair3* and *zep1* mutants. (A) Leptotene, faint HEI10 foci and bright spots are detected in *pair3*. Arrow indicates HEI10 spot. (B) Zygotene, faint HEI10 foci in *pair3* become diffused. (C) Pachytene, bright spot in *pair3* become diffused. (D) Zygotene, HEI10 consistently appears as foci in most *zep1* cells. (E) Zygotene, short HEI10 lines are found in *zep1* cells that contained compact chromosomes. (F) Late pachytene, *zep1* shows an increased number of HEI10 prominent foci. Scale bars, 5 µm.

To examine whether homologous pairing of chromosomes was required for HEI10 function, fluorescence immunolocalization studies were carried out in rice *pair3* mutant. PAIR3 is an axis-associated protein. Mutation of *PAIR3* resulted in the loss of homologous chromosome pairing [40]. In the *pair3-1* mutant, a few faint HEI10 foci (average 105, range 73–175, n = 20) were found on the chromosomes at leptotene. In addition, some cells also contained bright HEI10 spots (average 1.2, range 0–3, n = 25) (Figure 9A). During zygotene, the faint foci became diffused (Figure 9B). Similarly, the bright spots also became diffused and gradually disappeared at pachytene (Figure 9C). These results suggested that homologous pairing is required for proper HEI10 localization.

To characterize the effect of the synapsis mutation on HEI10 localization, immunolocalization studies were also performed using antibodies against REC8 and HEI10 on meiocytes of the *zep1-1* mutant. Synapsis is completely disrupted in the absence of ZEP1 [25]. In the *zep1-1* mutant, the localization of HEI10 was indistinguishable from WT during leptotene. However, from zygotene to early pachytene, when the HEI10 foci began to elongate and form linear signals along the full length of the homologous chromosomes in WT, in most *zep1-1* cells HEI10 signals appeared consistently as distinct foci on chromosome axes (average 388.2, range 368–430, n = 20) (Figure 9D). We also noted some short linear signals in 1.0% of *zep1-1* cells that contained compact chromosomes (Figure 9E). Although linear signal formation was severely disrupted in *zep1-1* mutants, prominent foci appeared normal on chromosome axes and linear signals were never found in late pachytene cells (Figure 9F), indicating that the formation of prominent foci is independent of linear signals. The mean number of HEI10 prominent foci was 36.2 in *zep1-1* (n = 36, range 28–46) compared to 24.5 for WT PMCs, showing that prominent foci increased by approximately 50% due to the mutation of *ZEP1*.

**Figure 10:**
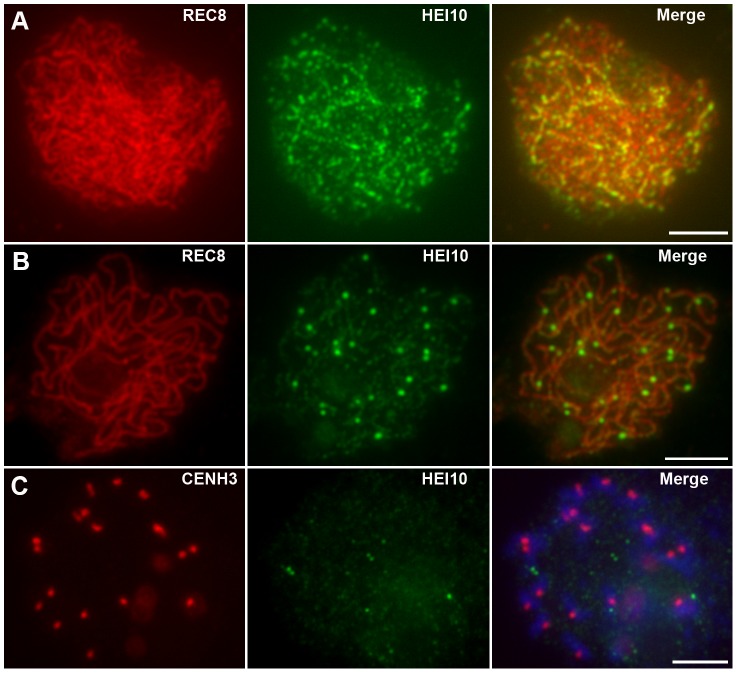
The localization of HEI10 in the *mer3* mutant. (A) Late leptotene, HEI10 foci localize normally. (B) Late pachytene, prominent HEI10 foci form normally. (C) Diakinesis, some prominent HEI10 foci locate on chromosomes while some disassociate from chromosomes. Chromosomes are stained with CENH3 signals (red) and DAPI (blue). Scale bars, 5 µm.

We next wanted to determine if the localization of HEI10 is affected by MER3, the mutation of which resulted in a similar reduction of CO number. In *mer3* mutants, HEI10 foci also appeared at early leptotene (Figure 10A). At late leptotene, a mean of 296.5 HEI10 foci are observed (n = 10, range 220–340). The localization of HEI10 at zygotene and pachytene was indistinguishable from that of WT rice. An average of 24.4 (n = 36, range 17–30) prominent foci were clearly detected in late pachytene cells (Figure 10B). During diakinesis, few HEI10 foci randomly distributed in the nucleoplasm and some foci also located on the remaining bivalents (average 2.9, range 0–7, n = 25) (Figure 10C). Based on those observations, it seems conceivable that in the absence of MER3, HEI10 foci localized normally until pachytene but most of them were not maintained at the later stages.

## Discussion

### HEI10 is required for normal CO formation in rice

The *HEI10* gene was first isolated from humans and it was demonstrated that the molecule played a role in the mitotic cell cycle [34]. Further analyses in mice revealed its role during meiosis. It was shown in mice that mutation of *HEI10* resulted in high prevalence of univalent chromosomes during metaphase I, which finally leads to a sterile phenotype. These data demonstrate that HEI10 is required for CO formation in mice [35]. Consistent with this, in the present study we show that the mutation of the *HEI10* gene in rice led to the occurrence of a large number of univalent chromosomes, suggesting that also in rice HEI10 is essential for reciprocal recombination between homologous chromosomes. In *hei10* mutants, only about 31% chiasmata were maintained. This number is similar to that of *mer3* mutants, in which about 28% chiasmata were remained. Thus, the close number of residual chiasmata indicates similar contributions of HEI10 and MER3 during CO formation.

Previous studies revealed that there are at least two classes of CO which occur in budding yeast and *Arabidopsis* [5], [22], [24]. Investigations of *mer3* mutants in rice showed that apart from a reduced number of chiasmata, the remaining chiasmata distributed randomly among cells, suggesting that rice might also have at least two kinds of COs: one that appears to be sensitive to interference, whereas the other one is not [20]. In this study, the residual chiasmata in *hei10* mutants displayed a similar random distribution among cells. Considering the close number and similar random distribution of residual chiasmata noted in *mer3* and *hei10* mutants, it is likely that *HEI10* and *MER3* may act in the same CO pathway, namely an interference-sensitive pathway.

In budding yeast, double *zmm* mutants and respective single mutants show nearly the same reduction of CO number [5]. Similar observations were also reported during the investigation of *Arabidopsis zmm* mutants [21], [27], [29]. In rice, although MER3 and HEI10 seem to be involved in the same CO pathway, the mean number of chiasmata in *mer3hei10* was significantly reduced compared to either single mutant. One possible explanation for the observation is that MER3 and HEI10 in rice might not be functionally equivalent with respect to forming class I CO. Thus, mutation of either gene would not completely disrupt all class I COs. In addition, recombination intermediates of rice might be more stable than that of budding yeast and *Arabidopsis*. So, in budding yeast and *Arabidopsis*, mutation of either *ZMM* gene might disrupt all class I recombination intermediates. In contrast, in rice, a few class I recombination intermediates might not be disrupted by the mutation of either *MER3* or *HEI10*.

Besides a reduced chiasma frequency, *hei10* also showed a precocious segregation of sister chromatids of some univalents at anaphase I. Such defects were also reported in other rice meiotic mutants, such as *pair1*, *pair3* and *mer3* mutants. Therefore, it is probable that the defects in *hei10* are caused by the indirect abnormalities of univalent sister kinetochores or prolonged metaphase/anaphase, rather than the direct effect of the absence of the HEI10 protein.

### HEI10 localizes independently from MER3 at leptotene

Using an antibody raised against HEI10, Western blot experiments showed that in mice HEI10 expression was low in *mei1*, in which meiosis arrest before the pachytene stage due to failed DSB formation and asynapsis. In addition, HEI10 protein was visible from early-mid pachytene. Based on those results, the authors speculated that HEI10 was not involved in partitioning of DSBs to the CO pathway, but may be required for processing CO recombination intermediates [41]. In rice, immunostaining experiments showed that HEI10 appeared in early leptotene, and exhibited high co-localization with MER3. This suggested that in rice HEI10 might act cooperatively with MER3 to process recombination intermediates in the early stages of meiotic prophase. In *mer3* mutants, the early localization of HEI10 was not affected. Additionally, in the *hei10* mutant, MER3 localized normally. Together these observations suggest that at the early stages of meiosis localization of MER3 and HEI10 is independent from each other.

### HEI10 extends along the chromosomes in the wake of synapsis

During zygotene, the stage at which synapsis occurs, HEI10 signals were stretched into linear arrays of dots or short lines. Interestingly, those linear HEI10 signals were distributed alongside elongated ZEP1 signals suggesting HEI10 progresses along the chromosomes during synapsis initiation in WT. In *zep1* mutants, linear HEI10 signals were rarely observed which suggested that ZEP1 plays an important role in HEI10 progression along homologous chromosomes. Similar pattern of meiotic proteins were described in *C. elegans* where ZHP-3, the ortholog of Zip3 in budding yeast, localized along the length of the SC in early pachytene. In SYP-1-depleted nuclei, however, ZHP-3 did not form lines at the pachytene stage. It was, therefore, concluded that the proper localization of ZHP-3 is dependent on the formation of a mature SC with incorporated central elements permitting ZHP-3 localization along the SC [32], [33]. However, in rice short linear HEI10 signals were still detected in a small number of *zep1* meiocytes which contained compact chromosomes. This indicated that the observed defects might be an indirect rather than a direct effect of the lack of SC. Previous studies suggested that close juxtaposition of two axes might be the only requirement for SC formation, which in turn would bring the paired homologs into an even closer juxtaposition [42]. By analogy, we postulate that the extension of HEI10 might only require the close juxtaposition of two axes rather than SC per se. Alternatively, it is also possible that an unknown intermediate protein plays the proposed role and that HEI10 positioning is assisted by the intermediate protein. In the case of compact *zep1* cells, the distances between chromosome axes are relatively closer, so linear HEI10 proteins may be partly assembled. Of course we cannot exclude the possibility that those short linear signals might merely be simple aggregates of HEI10 foci in *zep1*. Previous studies have shown that the rice *zep1* mutant retains CO formation and successful equal homolog segregation, which renders it difficult to unravel the exact role of SC in rice [25]. Thus, the real effect(s) of HEI10 localization along the SC remains unknown and needs further investigation.

### HEI10 prominent foci from late pachytene to diakinesis suggest class I COs

Recombination nodules were originally identified as electron-dense ovoid structures associated with SCs. Recombination nodules have been classified into two classes: early nodule (EN) and late nodule (LN) [43]–[45]. ENs have been postulated to correspond to initial recombination sites, whereas LNs are believed to indicate COs that are assumed to mature into chiasmata. ENs are more numerous and only a subset of them is likely to be converted into LNs [6], [44]–[46]. In budding yeast, ZMM foci correspond to LNs and are considered to mark final class I COs sites [6], [19]. In *C. elegans*, the Zip3 homolog ZHP-3 appeared in prominent foci and localized to CO sites in late pachytene and diplotene [33]. In mice, foci of MLH1 and MLH3 at the pachytene stage were used to mark the positions of meiotic COs which will subsequently develop into chiasmata [47], [48]. Consistent with this, in *Arabidopsis*, foci of MLH1 and MLH3 were used to indicate future CO sites [49]. Similarly, in the study presented here prominent foci of HEI10 were observed on the chromosomes of rice from late pachytene, which suggests that, apart from its important roles during early stages, HEI0 protein may play an additional role at the late stages. The average number of prominent HEI10 foci was about 2.0 per bivalent. The number approximately matches the expected number of COs per homolog. In addition, obvious interference among HEI10 bright foci on the shortest chromosomes was detected. Furthermore, we observed that the foci localized to the sites of chiasma occurrence during diakinesis. These results lead us to suggest that the bright foci of HEI10 in the late pachytene stage correspond to LNs, and mark chiasmata at diakinesis. Therefore, HEI10 is the first protein demonstrated to mark COs in rice. As discussed above, HEI10 seems to be involved in the interference-sensitive pathway, and therefore, approximately 24.3 foci in WT may be characteristic of class I COs.

Counting the number of CO by the shape of metaphase I bivalents showed that WT rice contains 20.6 COs per cell. It has been recognized that this method slightly underestimates the frequency of chiasmata because multiple COs, which frequently occur in WT chromosomes, were not counted [22]. Indeed, an analysis of a double haploid population revealed that about 28.3 COs coexisted in WT rice [50]. If the number of CO was universal in different background plants, it would be expected that approximately 85.9% of COs are formed via the interference-sensitive pathway. This is similar to values found in yeast and *Arabidopsis*, in which about 85% of COs were derived from interference-sensitive pathway [5], [21], [23]–[24], [27].

In *zep1* mutants, the distribution of HEI10 was severely affected but the prominent foci formed normally. This indicated that the formation of prominent HEI10 foci was independent of synapsis and HEI10 elongation. Previous analysis of *zep1* mutants revealed a tendency to contain increased numbers of COs [25]. However, the fluffy chromosomes of *zep1* mutants during diplotene and metaphase I prevented the counting of chiasmata. Here, the analysis of prominent HEI10 foci in the *zep1* mutant revealed an increase of COs from 24.5 to 36.2. Hence, it is likely that in *zep1* mutants class I COs are increased approximately 1.5-fold compared to that in WT. Of course, further genetic analysis is needed to determine the exact number.

### CO designation may occur independent of ZMM proteins in rice

In yeast *zmm* mutants, it has been shown that DSBs and NCOs form normally while SEIs and dHJs were both defective. These results implied that CO designation occurs prior to and independent of ZMM function in budding yeast [5]. This implication is supported by the finding that yeast *msh4* and *zip1* mutants showed a nearly normal distribution of ZIP2 foci, which mark LNs at the pachytene stage [6].

As many aspects of meiotic recombination seem to be conserved in plants and budding yeast, it is tempting to suppose that the CO/NCO decision also occurs at an early stage in plants. Nevertheless, direct evidence for this is lacking [51]. In this study, in contrast to *pair3* and *zep1* mutants, the rice *mer3* mutant showed a nearly normal number of prominent HEI10 foci at late pachytene. Although it is unclear when CO designation is made in rice, the nearly normal distribution of HEI10 foci at late pachytene in *mer3* mutants indicated that the CO designation in *mer3* may be made normally and may still be maintained until late pachytene. These results also suggest that the CO designation may be imposed independent of MER3 function in rice. However, most foci did not persist on chromosomes to diakinesis although they formed normally at late pachytene. Therefore, due to failed processing of recombination intermediates, most class I CO-designated sites may lose their designation after the pachytene stage and may finally be repaired as NCOs or toward the sister chromatid. Similar observations were reported from an *Arabidopsis* ZMM mutant, *ptd*, where nearly normal numbers of LNs were observed at pachytene [31] suggesting that the mechanisms to designate COs are potentially conserved among different organisms.

### HEI10 may be the ortholog of Zip3 and ZHP-3

With the exception of Zip3, all other ZMM orthologs have been identified and investigated in plants. Here, by isolation of the rice *HEI10*, we proposed a close relationship among HEI10, ZHP-3 and Zip3. Firstly, rice HEI10 show significant similarity to mammalian HEI10. Reciprocal protein BLAST searches revealed that rice HEI10 and *C. elegans* ZHP-3 are the closest relatives in their respective species and are probably orthologs. Secondly, HEI10, ZHP-3 and Zip3 contain two similar domains, namely an N-terminal RING finger domain and a coiled coil domain. Bioinfomatic analysis and *in vitro* experiments have revealed that Zip3 may be a SUMO E3 ligase [13]–[14], [52]. A recent study in mice suggested that HEI10 has SUMO E3 ligase function in addition to its reported E3 ubiquitin ligase activity [41]. Thirdly, HEI10, ZHP-3 and Zip3 are probably all involved in class I COs. Mutation of either gene in their respective species showed similar defects in class I COs. Additionally, the dynamic localization pattern of HEI10 is similar to that of ZHP-3. Taken together, all these evidences support the idea that HEI10 is the most likely ortholog of ZHP-3 and Zip3 in rice, although other proteins belonging to the Zip3 family cannot be excluded. In contrast to Zip3, both HEI10 and ZHP-3 exhibit a similar dynamic localization pattern, implying that the proteins may evolve new functions during meiotic recombination in multicellular organism. In *C. elegans* ZHP-3 was required for SC asymmetrical disassembly and normal bivalent structure [33]. Such defects were not observed in rice, implying probable diversification of Zip3 homologs even among multicelluar organisms.

In this study, the dynamic location of HEI10 during meiosis in rice not only indicated early recombination events at leptotene, SC formation at zygotene and pachytene, but also marked late recombination events from late pachytene to diakinesis. HEI10 protein has been suggested to have E3 ligase activity that is involved in ubiquitinylation and/or SUMOylation processes, which have a wide variety of protein regulatory roles [41], [53]. Thus, we propose that HEI10 might continuously promote class I CO formation through modification of a series of meiotic protein targets, including EN components, SC proteins and LN components.

## Materials and Methods

### Plant materials

Two *hei10* mutants were isolated in *japonica* rice varieties, Wuxiangjing 9 and Nipponbare, independently. The heterozygous plants were crossed with 9311 (*indica* variety) individually to make mapping populations. All plants were grown in paddy fields.

### Molecular cloning of *HEI10*

To fine map *HEI10*, STS markers were developed based on sequence differences between *japonica* variety Nipponbare and *indica* variety 9311 according to the data published in http://www.ncbi.nlm.nih.gov. The primer sequences of STS markers are as follows: P1 (TCCTAGTGCTTGCTAACC and AGCACCTACGGTAAGCAGG), P2 (GGTGACTACGCCAACTTCGG and CTCAAGCGCAACTATTCGGG), P3 (TCACCTCCTCTCCACCTTAGA and AAGCCAAGGCGACTGTTG), P4 (AAGACACGGAGGAGGAGAAC and CTTTTAACTTGGCTTTGGCT), P5 (AGCTACATCGATCTCAGTT and CTGCATTTGCACCGTTTGTT), P6 (CGACAAGAAGCATCAAATCA and ATGATGAGGAGATTTTGTTC), P7 (GATGAGTTGTGAGAAAATACT and CATAGTCATTAGCTGCAGTGT), P8 (TGCACTTGACATGATGAGTT and CGCTTCAGTAATCATAGTCA).

### Molecular cloning of *HEI10* cDNA

Total RNA was extracted from rice young panicles (5–8 cm) using TRIZOL reagent (Invitrogen) as described by the supplier. 3 µg RNA was reverse-transcribed with Adaptor-T(18) primer (CTGATCTAGAGGTACCGGATCCTTTTTTTTTTTTTTTTTT) using the superscript III RNaseH reverse transcriptase (Invitrogen). For 3′RACE, two rounds of PCRs were carried out using primers Adaptor primer (CTGATCTAGAGGTACCGGAT), gene specific primer RT-F1 (GGAAGCTTGATGAGATGTAT) and primer RT-F2 (GCAGCGACCCTCTGAGAC). The cDNA ORF was also confirmed using primers FOR (GCAAAGCAATTTTGCCATTC) and END (ACAGTTAGCAAGTCCATGAC).

### Antibodies

The REC8, MER3 and ZEP1 polyclonal antibodies used in this study were described before [20]. To generate the antibody against HEI10, a 919 bp fragment of *HEI10* cDNA was amplified using primers AB1F (AAGGATCCATGAAGTGCAATGCTTGCTG) and AB1R (AACTCGAGGACATTACGTGAACAT). The fragment was ligated into the expression vector pGEX-4T-2 (Amersham) digested with *Bam*HI-*Xho*I. The expression vectors were transformed into BL21 (DE3) and were induced by addition of 0.2 mM IPTG to the culture medium. The fusion peptides were accumulated predominantly in the form of inclusion bodies. After gel electrophoresis and Coomassie blue staining, the protein bands were sliced and ground into powders. Then, the powders were used to raise antibodies in rabbit and mouse, respectively. The antibodies did not give signals in *hei10* mutants, indicating the specificity of the antibodies.

### Cytology

Young panicles of at meiosis stage were harvested and fixed in Carnoy's solution (ethanol∶glacial acetic, 3∶1) for at least 24 h at room temperature. Microsporocytes were squashed with a needle in an acetocarmine solution and covered with a coverslip. Slides with chromosomes were frozen in liquid nitrogen. After soaking in liquid nitrogen and removing the coverslip, the slides were dehydrated through an ethanol series (70%, 90%, and 100%). Chromosomes were stained with DAPI in an antifade solution (Vector Laboratories). Immunofluorescence studies were carried out as previously described [20].

### Computational and database analysis

The gene structure schematic diagram was drawn using GSDS (http://gsds.cbi.pku.edu.cn/index.php) and further edited with Adobe Illustrator CS2. Protein sequence similarity searches were performed at the NCBI (http://www.ncbi.nlm.nih.gov/BLAST). The alignment of amino acid sequences was performed with the MEGA3.1 software (http://www.megasoftware.net/), and further modified using Alignx module of Vector NTI Advance 9.0 (Invitrogen). Domain searches were performed using the HMMER-based SMART Web site (http://smart.embl-heidelberg.de/).

### Interference measurement

We measured the lengths of SCs (AEs) and the positions of HEI10 bright foci on chromosome using the IPLAB software. The data were collected and exported into Microsoft Excel for analysis. We took the interference parameter *ν* of the gamma model as a measure for the strength of interference among HEI10 bright foci on the shortest chromosome in rice PMCs [38], [39]. We obtained the estimate of *ν* by fitting the observed relative frequencies of inter-HEI10 foci distances to the gamma distribution using the MATLAB software package.

### Accession numbers

The GenBank (http://www.ncbi.nlm.nih.gov/GeneBank) accession number for HEI10A and HEI10B cDNAs are JQ624877 and JQ624878, respectively. The accession number for *Arabidopsis* is NP_175754, Human NP 878271, Mouse NP_001104589. The accession number for the budding yeast Zip3 is NP_013498, for *C.elegans* ZHP-3 is NP_492311.

## Supporting Information

Figure S1 The rice *hei10-1* mutant phenotype. (A) Comparison of a WT plant (left) and a *hei10-1* mutant plant (right). (B) Comparison of a WT panicle (left) and a *hei10-1* mutant panicle (right). (C) Fertile pollen grains in a WT plant. (D) Sterile pollen grains in a *hei10-1* mutant plant. Scale bars: 50 µm.
(TIF)

Figure S2 Alignment of HEI10 homologues. Identical amino acids are shaded in black whereas similar amino acids are shaded in gray. The black box indicates the absent amino acids in HEI10B protein sequence. Os = *Oryza sativa*; At = *A. thaliana*; Hs = *H. sapiens*; Mm = *M. musculus*; Ce = *C. elegans*; Sc = *S. cerevisiae*.
(TIF)

Figure S3 Meiosis in the WT. (A) Pachytene. (B) Diakinesis. (C) Metaphase I, the right two bivalents are treated as having one chiasma while other bivalents are treated as having two chiasmata. (D) Telephase I. (E) Prophase II. (F) Tetrads. Scale bars: 5 µm.
(TIF)
