# Supplementary figures and images for: The Role of Rice HEI10 in the Formation of Meiotic Crossovers
Source: PLoS Genet. 2012 Jul 5;8(7):e1002809. doi: 10.1371/journal.pgen.1002809 (PMC3390396; doi:10.1371/journal.pgen.1002809)

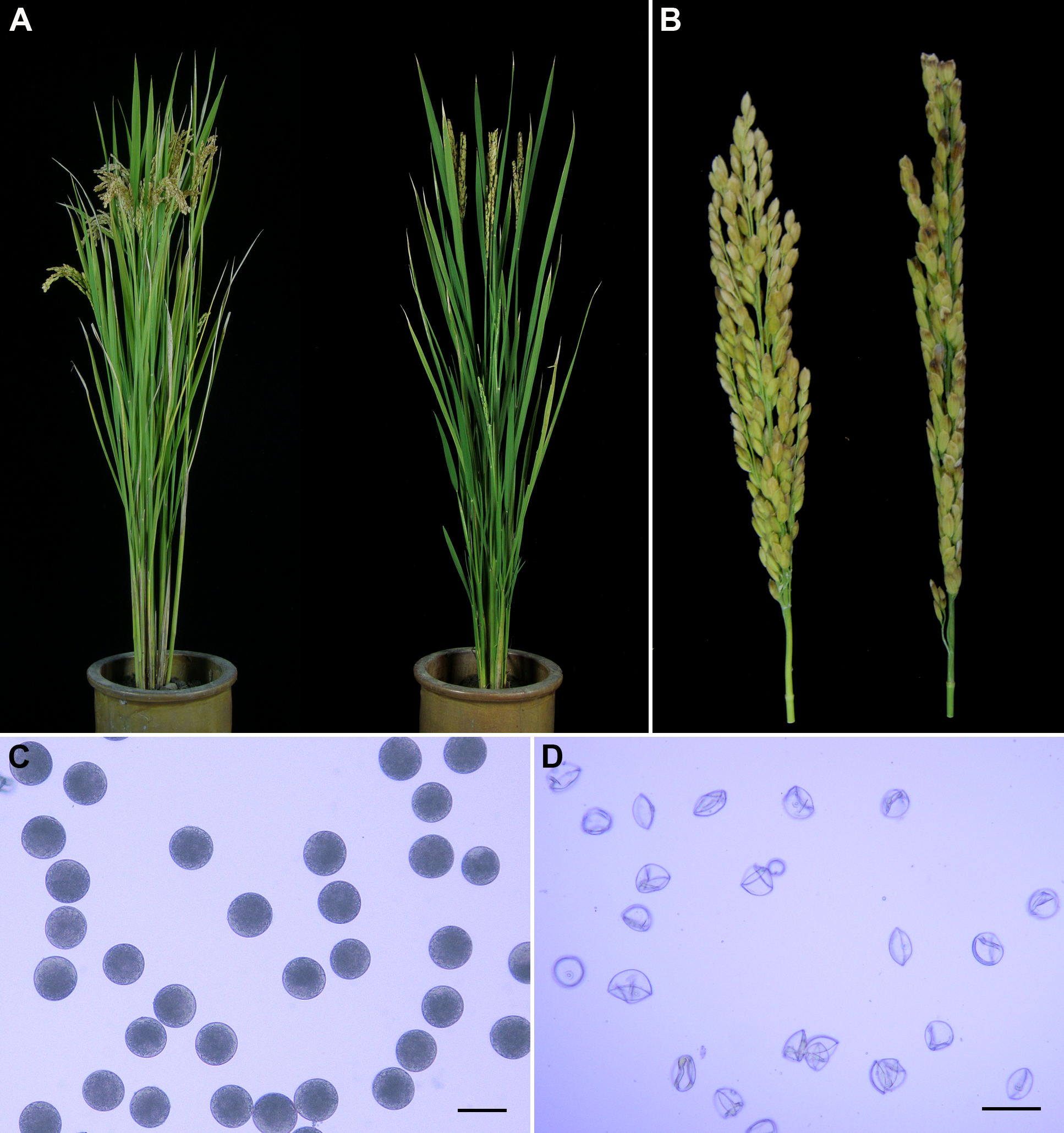

Supplement: Figure S1 — The rice hei10-1 mutant phenotype. (A) Comparison of a WT plant (left) and a hei10-1 mutant plant (right). (B) Comparison of a WT panicle (left) and a hei10-1 mutant panicle (right). (C) Fertile pollen grains in a WT plant. (D) Sterile pollen grains in a hei10-1 mutant plant. Scale bars: 50 µm. (TIF) [file pgen.1002809.s001.tif]

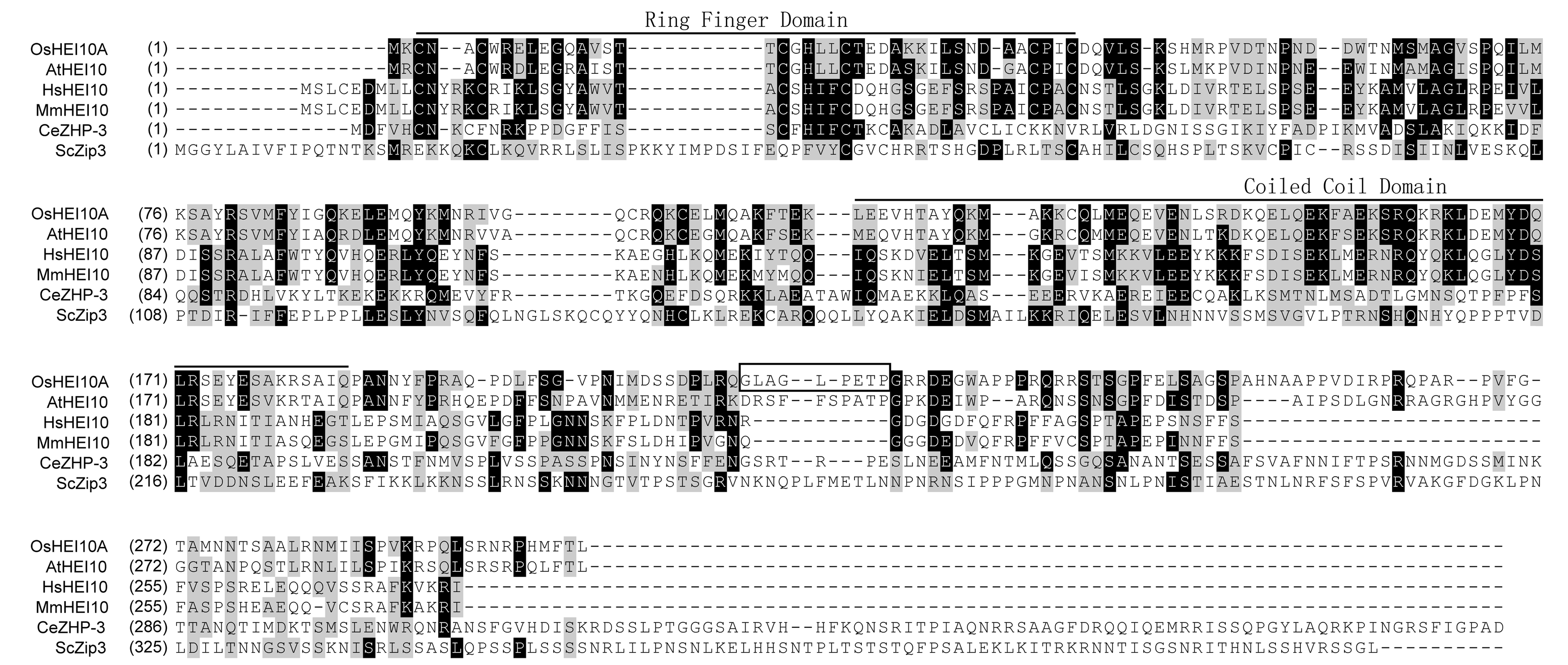

Supplement: Figure S2 — Alignment of HEI10 homologues. Identical amino acids are shaded in black whereas similar amino acids are shaded in gray. The black box indicates the absent amino acids in HEI10B protein sequence. Os = Oryza sativa; At = A. thaliana; Hs = H. sapiens; Mm = M. musculus; Ce = C. elegans; Sc = S. cerevisiae. (TIF) [file pgen.1002809.s002.tif]

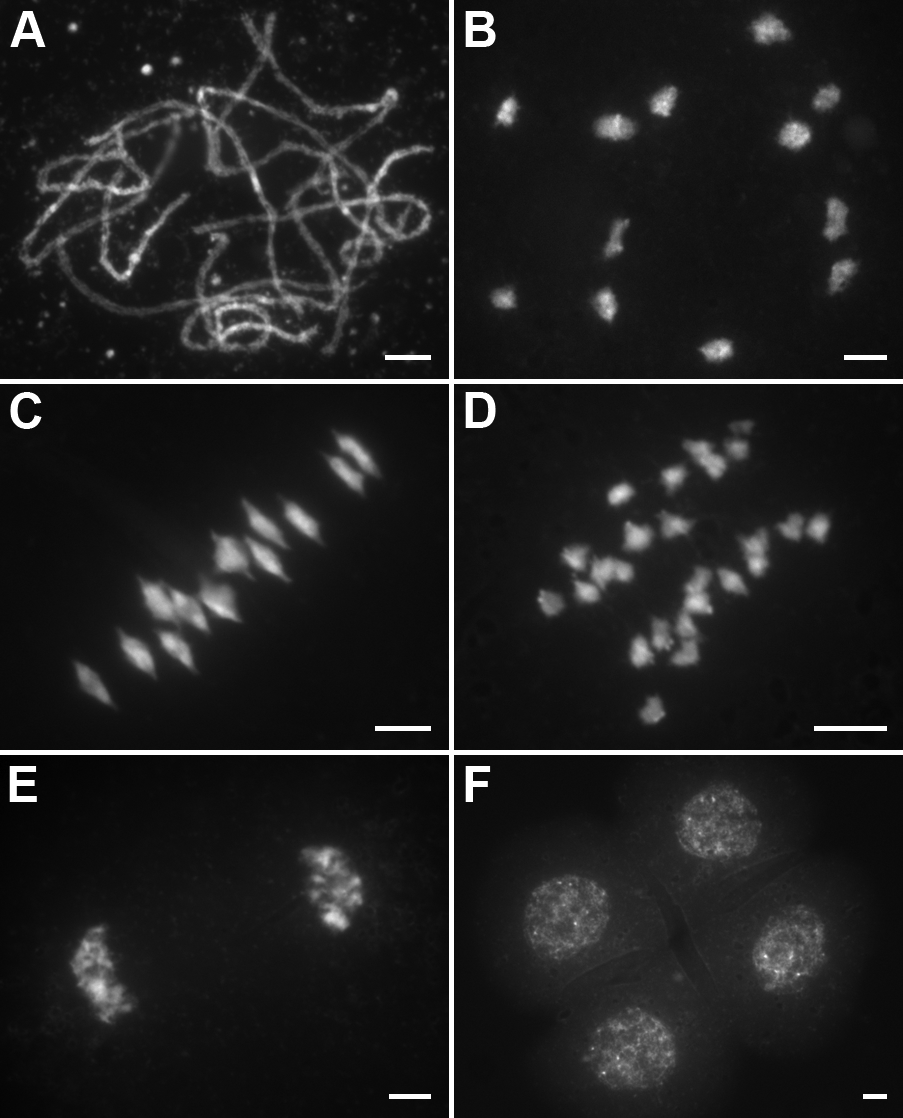

Supplement: Figure S3 — Meiosis in the WT. (A) Pachytene. (B) Diakinesis. (C) Metaphase I, the right two bivalents are treated as having one chiasma while other bivalents are treated as having two chiasmata. (D) Telephase I. (E) Prophase II. (F) Tetrads. Scale bars: 5 µm. (TIF) [file pgen.1002809.s003.tif]
